# Removal of ammonium ion from aqueous solutions by using unmodified and H_2_O_2_-modified zeolitic waste

**DOI:** 10.1038/s41598-019-55906-0

**Published:** 2020-01-15

**Authors:** Danutė Vaičiukynienė, Agnė Mikelionienė, Arūnas Baltušnikas, Aras Kantautas, Algirdas Radzevičius

**Affiliations:** 1 Faculty of Water and Land Management Agriculture Academy, Vytautas Magnus University, Studentu st. 11, Akademija, 53361 Lithuania; 2 Laboratory of Materials Research and Testing, Lithuanian Energy Institute (LEI), Breslaujos st. 3, Kaunas, 44403 Lithuania; 3 Faculty of Chemical Technology, Kaunas University of Technology, Radvilenu st. 19, Kaunas, 50254 Lithuania

**Keywords:** Chemical engineering, Ceramics

## Abstract

In the petroleum industry during a catalytic cracking process, the used zeolitic catalyst becomes waste. This article investigated the sorption capacities of ammonium ions from aqueous solutions onto the previously mentioned zeolitic waste by batch experiments. Three types of zeolitic waste were used: unmodified zeolitic waste with two different particle size distributions and H_2_O_2_-modified zeolitic waste. Several techniques, including X-ray diffraction (XRD) analysis, Fourier transform infrared spectroscopy (FT-IR), Brunauer-Emmett-Teller (BET) multilayer adsorption theory measurements, and X-ray fluorescence analysis (XRF) were used to demonstrate experimentally that the zeolitic waste could be used as a sorbent for the water decontamination of NH_4_^+^ ions. The morphology of zeolitic waste investigated by scanning electron microscopy (SEM) revealed particles with a spherical shape. The nitrogen adsorption–desorption isotherms showed an isotherm mixture of types I (pure microporous) and IV (mesoporous). This suggested that the investigated zeolitic materials were mesoporous (4.84 nm) and microporous (0.852 nm), as well as containing slit/cylindric pores, according to a quench solid density functional theory (QSDFT) adsorption branch model. Zeolitic waste from the oil industry showed good NH_4_^+^ sorption properties (removal efficiency of 72%), thus becoming a potential adsorbent to be used in the treatment of contaminated aqueous effluents polluted with ammonium ions. Simultaneous waste and water decontamination can be achieved, providing a new tool and enhanced capabilities for environmental remediation.

## Introduction

Ammonium (NH_4_^+^) is one of the common form of reactive nitrogen in wastewater^1^, and its contamination in fish causes serious health problems due to its high toxicity. The technologies for ammonia removal from wastewater are based on physicochemical and biochemical treatment methods^2^. One of these treatment methods is adsorption, which is a low-cost process. Different adsorbents, such as wheat straw biochars, pine sawdust or zeolites, can be effective in adsorbing ammonium in wastewater^3–13^. Yang *et al*.^4^ used some natural adsorbents such as pine sawdust and wheat straw biochars for the ammonium removal in wastewater. It was concluded that biochars can be efficient absorbents for NH4^+^ removal from aqueous solutions. Tian *et al*.^5^ synthesized a new sorbent using modified coal cinders and zeolite powders. This sorbent has been shown to be a good material for the removal of ammonia nitrogen. The removal efficiency of ammonia nitrogen was 67.3% and 71.3%. The inflow concentrations of ammonia nitrogen were obtained under the experimental conditions where chemical oxygen demand under a water flow of 10 L/h. Yin *et al*.^6^ used NaCl-modified clinoptilolite as adsorbent for the nitrogen removal. Results in a current batch study provided that the maximum ammonium sorption capacity of clinoptilolite was in the range of 6.64 to 7.27 mg. N/g. The work reported by Sánchez-Hernández *et al*.^7^ evaluated the use of NaP1 zeolite for the removal of ammonium from aqueous solutions. The experiment of ammonium removal was conducted by the batch experiments. This zeolite was obtained from a hazardous Al-containing waste. These results indicate that the duration of the NH_4_^+^ uptake on NaP1 was fast. Ammonium was removed within 15 minutes with high sorption capacity (37.9 mg/g). Huo *et al*.^8^ reported that a salt and a thermally modified clinoptilolite had the potential effectively to remove ammonia. This modified zeolite had rapid adsorption and slow balance characteristics. An entropy effect is very important in the adsorption process. Xue *et al*.^9^ examined different types of zeolites for ammonia removal in high ammonia water. According to this experimental results it could be concluded that mordenite is suitable to remove ammonia from water.

Several studies have reported on the adsorption of ammonium ions by natural or synthetic zeolitic material adsorbents as well^10–13^. Zeolite - aluminosilicate hydrate minerals with a porous, three-dimensional crystal structure are considered an excellent ion-exchange material because of their high selectivity for NH_4_^+^ due to their microstructure^3^. An adsorbent of natural zeolites possesses a polar surface and is therefore able to attract ammonium ions quickly and effectively^2^. The removal of ammonia from water was carried out by using natural and synthetic zeolites. In this research three types of natural zeolites (clinoptilolite, mordenite and ferrierite), and synthetic zeolite A were used. The different forms of zeolites such as sodium, potassium and calcium forms were investigated^10^. It were concluded that natural zeolites show high selectivity for ammonium ions with respect to other monovalent ions despite the much higher theoretical exchange capacity of zeolite A. In the study^11^, the clinoptilolite was fused with sodium hydroxide prior to a hydrothermal reaction, and it was transformed to a modified zeolite Na–Y. The results were acceptable, showing that modified zeolite Na–Y exhibited a much higher uptake capacity compared with that of clinoptilolite. At an initial concentration of 250 mg/L NH_4_^+^, the ammonium ion uptake value of sample 2 was 19.29 mg/g NH_4_^+^ adsorbed, while that for sample 1 was only 10.49 mg/g^1^ NH_4_^+^ adsorbed. Markou *et al*.^12^ investigated zeolites which were synthesized from coal fly ash in sodium form and in potassium form as well. These zeolitic materials were used as sorbents for ammonium removal. According to this study the maximum sorption capacities were 109 ± 4 mg/g NH_4_ and 33 ± 1 mg/g NH_4_ for sodium form zeolite and potassium form zeolite, respectively. Wu *et al*.^14^ investigated novel granular adsorbents produced with zeolite powders and an Al–Mn binary oxide via a compression method for removing ammonium from wastewater. Kinetic adsorption results indicated a fast adsorption rate for NH_3_-N, and the maximum adsorption capacity of NH_3_-N was 12.9 mg/g via the Langmuir model.

There is some research related to improving aquaculture water quality using of natural or synthetic zeolites. Ammonia removal was conducted using an aquaponics-zeolite system^13^. Green mustards were used as aquaponic plants, and natural zeolites were modified using sodium chloride (0–5%). The aquaponic and zeolite adsorption method had a significant effect on a fish pond. Bergero *et al*.^15^ tested ammonia removal from aquaculture water in recirculating systems by using as adsorbent different types of zeolites under laboratory conditions. Natural zeolites such as phillipsite and clinoptilolite tuffs were beneficial in ammonia removal. Another type of natural zeolitic material (chabazitic tuff with a lower amount of zeolitic material (50%))_ had a lower cation exchange capacity for ammonia. It was concluded that, a lower temperature did not effected significantly the ion exchange capacity in any of the investigated zeolites. The results of this type fish farming water treatment would benefit in the design of a new nutrient purification tool. This water treatment method is close related with the sustainable management of aquaculture.

There are many articles on the removal of ammonium ions using different natural and synthetic sorbents, but information related to NH_4_^+^ sorption using zeolitic waste has not been found. In this study, the zeolitic waste from fluid catalytic cracking (FCC) catalysts was used as adsorbents for ammonium ions. Previous investigations have shown that this waste exhibits good removal properties for chromium removal^16^, encapsulates heavy metals^17^, and is suitable for recycling phosphogypsum (it adsorbs harmful admixtures)^18^.

In spite of existing reports on NH_4_^+^ removal using natural or synthetic zeolites, the present study suggests the use of zeolitic waste as a sorbent material. Thus, in this way, it will be possible to reuse zeolitic waste and save natural resources when natural zeolites are used as sorbents. Furthermore, using a zeolitic waste sorbent for NH_4_^+^ removal would be more economical than previous applications where natural or synthetic zeolite were used.

The aim of this study is to determine the NH_4_^+^ sorption removal efficiency of three different types of zeolitic waste from the oil industry. Two types were unmodified zeolitic waste with two different particle size distributions, and the third type was a H_2_O_2_-modified zeolitic waste.

## Experimental

### Experimental techniques

The chemical composition of zeolitic waste was determined by X-ray fluorescence spectrometry (XRF) on a Bruker X-ray S8 Tiger WD using a rhodium (Rh) tube, an anode voltage *U*_*a*_ up to 60 kV, and an electric current *I* up to 130 mA. The pressed samples were measured in a helium atmosphere. Measurements were performed using a SPECTRA Plus QUANT EXPRESS method^19^.

X-ray diffraction analysis (XRD) of the materials was performed using a D8 Advance diffractometer (Bruker AXS) operating at a tube voltage of 40 kV and a tube current of 40 mA. The X-ray beam was filtered with a Ni 0.02-mm filter to select a CuKα wavelength. The specimens were scanned over a 2θ range from 3° to 70° at a scanning speed of 6° min^−1^ using a coupled two theta/theta scan type^20^.

A laser particle size analyzer (CILAS 1090 LD) determined the particle size distribution and the specific surface area of the investigated materials in intervals from 0.04 to 500 μm. The distribution of solid particles in the air stream was 12–15 wt.%. Compressed air (2500 mbar) was used as a dispersing phase. The measuring time was 15 s^21^.

The microstructures of the three types of zeolitic waste were studied by scanning electron microscopy. A high-resolution scanning electron microscope (ZEISS EVO MA10) was used for the research^22^.

The pH measurements of water suspensions were conducted by using a WTW pH 320 pH-meter.

Fourier transform infrared (FT-IR) spectrometry was conducted with a Perkin Elmer FT-IR System spectrometer. One milligram of the substance was mixed with 200 mg of KBr and compressed in a forming press under vacuum for the IR analysis^23^.

The textural parameters of the samples were determined by nitrogen adsorption–desorption isotherms at −196 °C (77 K) using a Quantachrome Autosorb-iQ-KR/MP automated gas sorption analyzer. Prior to the analysis, the powder samples were outgassed under vacuum at 105 °C for 3 h. The specific surface area was calculated by using the BET (Brunauer–Emmett–Teller) equation. The pore size distribution was determined by applying the density functional theory (DFT). The total pore volume was measured from the adsorption isotherm by the uptake of nitrogen at a relative pressure of p/p_0_ = 0.99. All calculations were performed by an ASiQwin (Version 2.0) program developed by Quantachrome Instrument^24^.

The evaluation of the NH_4_^+^ concentration was evaluated according to the Nessler method, and the ammonium (NH_4_^+^) concentration was measured by a UV-VIS spectrophotometer (SPECORD PLUS). An ammonium chloride (NH_4_Cl) salt was used to make the NH_4_^+^ solutions.

The ion exchange of NH_4_^+^ ions on the zeolite was carried out using a batch method. The batch experiments were conducted with 2 g of adsorbent in 200 ml of solutions with 1 mg/L NH_4_^+^ and 10 mg/L NH_4_^+^. Then, all samples were left at 20 °C under static conditions for 1 h, 12 h, 24 h, 48 h and 72 h. Ammonium standard solution (1000 mg/L NH_4_^+^) and deionized water were used for the preparation of the initial ammonium solutions. The pH of the solutions was also evaluated.

The removal efficiency (%) and the amount of exchanged NH_4_^+^ ion (*q*_*e*_) by the zeolite were evaluated using Eqs. (1) and (2), respectively^25^:

1 $${\rm{Removal}}\,{\rm{efficiency}}( \% )=\frac{({C}_{0}-{C}_{e})}{{C}_{0}}\cdot 100 \% ;$$

2 $${q}_{e}=\frac{({C}_{0}-{C}_{e})V}{m}.$$

where *q*_*e*_ is the amount of exchanged ammonium ions (mg/g), *C*_0_ and *C*_*e*_ are the initial and equilibrium concentrations of ammonium in solution (mg/L), respectively, *V* is the solution volume (L), and *m* is the adsorbent weight (g).

In solution, the experiment for the amount of NH_4_^+^ was repeated at least three times. The mean value of the triple analysis was used to calculate the amount of NH_4_^+^ in solution, and the limit of error for the samples was lower than 5%.

The point of zero charge, pHpzc, of the investigated zeolitic waste was evaluated according to Nasiruddin *et al*.^26^. For this purpose, a pH drift method was used. Sodium chloride (0.01 M) was used as a background electrolyte. Initial solutions with pH values in a range from 2 to 9 were prepared by adding small amounts of 0.5 M HCl or 0.5 M NaOH solutions. Then, in each simulated solution (20 ml), 0.5 g of zeolitic material samples were added, and the samples were left to settle for 24 h at room temperature. The final pH of each solution was measured. The pHpzc of zeolitic materials was evaluated; if the initial pH of the solution was equal to the final pH of the solution, then that was considered the pHpzc^27^, and the charge on the surface was zero.

## Materials

Zeolitic catalysts are very important in the petroleum refining industry as fluid catalytic cracking (FCC) units. In this study, the waste FCC catalyst was used as zeolitic waste. After some time during processing, zeolite can be polluted with oil products such as coke, sulfur, vanadium and nickel. After that, it became unsuitable for use in oil technology. The quantity of this waste inevitably rises due to a rapidly expanding oil industry. The composition of these catalysts depends on the manufacturer and on the process that is going to be used.

Due to cation exchange capacity and molecular network properties, zeolites are widely used as adsorbents in separation and purification processes. Zeolitic waste was used for the sorption of ammonium ions. The general chemical composition of zeolitic waste was measured according to XRF and is presented in Table 1. In all zeolitic waste, silicon and aluminum prevail with small amounts of carbon, lanthanum, magnesium and titanium.

**Table 1:** Chemical composition of the zeolitic waste (XRF), wt. %.

Element	Zeo waste 1	Zeo waste 2	Zeo waste 3
Oxygen	54.51	54.22	52.37
Carbon	3.31	1.25	3.34
Aluminium	23.00	24.92	24.25
Silicon	16.18	15.77	16.34
Magnesium	0.44	0.91	0.82
Sulfur	0.15	—	0.19
Calcium	—	—	0.47
Sodium	0.07	0.02	0.09
Iron	0.81	1.17	0.69
Titanium	0.39	0.65	0.26
Lanthanum	1.15	1.09	1.18

All chemicals, such as ammonium chloride (NH_4_Cl), hydrogen peroxide (H_2_O_2_) and sodium hydroxide (NaOH) were analytical grade.

## Results and Discussion

### The modification of zeolitic waste by using hydrogen peroxide solution

Zeolites are often modified to improve their sorption capacity. There are various zeolite modification and activation methods. One method for zeolitic waste modification is to use hydrogen peroxide (H_2_O_2_). H_2_O_2_ is an active oxidizer and is commonly used to transform, immobilize or remove carbonaceous impurities from zeolites. Canli *et al*.^28^ concluded that zeolite activation with H_2_O_2_ can improve the zeolite sorption capacity. The oxidation process of H_2_O_2_ has also been investigated for the regeneration of sorption properties of hydrophobic zeolites^29,30^. According to Singh *et al*. and Koryabkina *et al*., one of the advantages of using hydrogen peroxide in regenerative processes is that the hydrogen peroxide decomposition products are oxygen and water, which are environmentally friendly.

Thus, the last type of zeolitic material was H_2_O_2_-modified zeolitic waste (Zeo waste 2). The zeolitic waste was added to a 15% H_2_O_2_ aqueous solution and left for 24 h at ambient temperature. After that solution was separated from zeolitic waste by filtration, it was dried at 100 °C for 24 h. The chemical composition of Zeo waste 2 is shown in Table 1. According to the chemical analysis of this material, the amount of carbon (technically coke) significantly decreased from 3.31% in the unmodified zeolitic waste to 1.25% in the H_2_O_2_-treated zeolitic waste. Furthermore, sulfur was not detected at all. Therefore, by treating the waste with H_2_O_2_ solution during oxidation reactions, it can eliminate oil product pollutants such as coke and sulfur as well.

**Figure 1:**
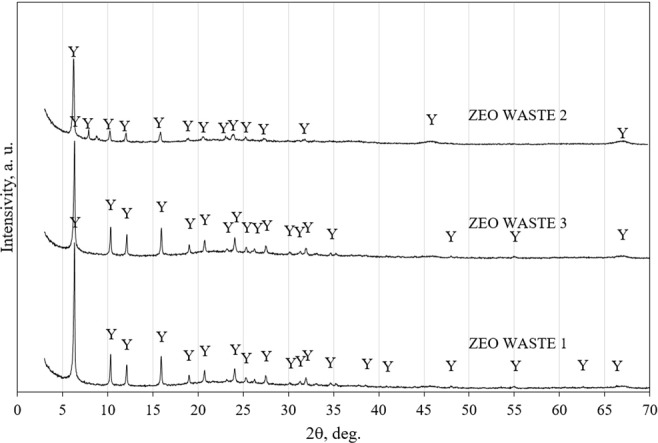
The X-ray diffraction patterns of zeolitic waste. Note: Y is zeolite Al_60.352_∙Si_139_∙O_371_._52_∙H_5.984_ (73-2313).

The mineral composition of the three types of zeolitic waste was evaluated by using XRD analysis (Fig. 1). This analysis showed that in all materials, similar minerals prevail. The mineral composition consisted of zeolite Y Al_60_._352_∙Si_139_∙O_371_._52_∙H_5_._984_. The main diffraction peaks belonged to this mineral and had main interplanar distances (d) of 1.399, 0.857, 0.731, 0.556, 0.429, and 0.370 nm.

Therefore, after the modification of zeolitic waste with a hydrogen peroxide solution, the mineral composition of the zeolitic waste did not change, but the amount of carbon significantly decreased, and sulfur was not detected in this material (Table 1).

### Particle size distribution and morphology of zeolitic waste

The particle size of zeolitic byproduct powder is very useful for predicting the main characteristics of ammonium sorption. The effect of particle size with the three different zeolitic waste products was evaluated. It is known that zeolitic powder size has a large influence on the sorption rate and maximum sorption capacity. Therefore, the ammonium sorption increased with decreasing zeolite particle size^31,32^.

**Figure 2:**
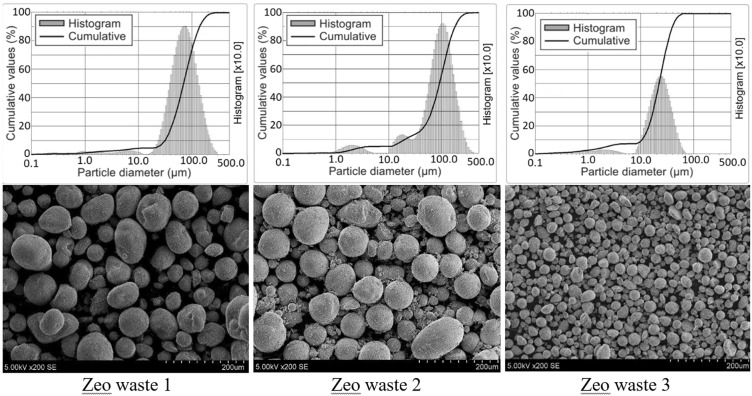
Scanning electron micrographs and particle size distribution of zeolitic waste powder.

Figure 2 demonstrates the particle size distribution of zeolitic waste powders and the microstructure of these powders. All three types of zeolitic waste powder had similar narrow particle size distributions. Zeo waste 1 had a mean particle diameter of 97.95 μm. Zeo waste 2 powder was similar to Zeo waste 1 with a value of 78.39 μm, and Zeo waste 3 had particles that were more than four times finer (mean diameter of 23.26 μm) compared with the powder from Zeo waste 1. In a catalytic cracker, a zeolite catalyst is used. In a closed system, the circulation of this catalyst occurs. After some time, the zeolitic catalyst becomes finer due to mechanical action, contact with hydrocarbons, water vapor, and the combustion process. Then, the zeolitic dust is separated by separators.

Table 2 demonstrates the particle size analysis characteristics of the three different samples. The particle size analysis showed that the zeolitic waste powder had a wide particle size distribution ranging from 177.81 μm to 19.80 μm and from 140.04 μm to 31.46 μm for Zeo waste 1 and Zeo waste 2, respectively. The narrower particle size distribution, ranging from 39.00 μm to 10.39 μm, was for Zeo waste 3.

**Table 2:** Characteristics of the powder mixtures obtained from particle size distributions.

Zeolitic waste	d90, μm	d50, μm	d10, μm	Mean diameter, μm
Zeo waste 1	177.81	90.91	19.80	97.95
Zeo waste 2	140.04	69.67	31.46	78.39
Zeo waste 3	39.00	22.02	10.39	23.26

The particle size distributions measured are in agreement with the SEM micrographs (Fig. 2b). In all three cases, the particles of the powders were spherical in shape^33^. Additionally, it could be observed that finer particles prevail in Zeo waste 3 compared with those in Zeo waste 1 and Zeo waste 2, which have similar sizes.

### The textural properties of zeolitic waste

The surface area and pore structure are important parameters for the ammonium sorption capacity of zeolitic waste. According to these parameters, it is possible to predict the main characteristic of ammonium sorption.

The surface area and pore structure of the three types of zeolitic waste were investigated by analyzing nitrogen adsorption-desorption isotherms. The BET specific surface area of the zeolitic particles, the specific surface area of the pores, the average pore size and the pore volume are presented in Table 3.

**Table 3:** N_2_ adsorption-desorption results of zeolitic waste.

Material	S_BET_, m^2^/g	Pore structure		
		S_Pore_, m^2^/g	Average pore size, nm	Pore volume, cm^3^/g
Zeo waste 1	137.69	138.43	0.852	0.187
Zeo waste 2	137.39	143.70	0.852	0.173
Zeo waste 3	138.89	130.08	0.852	0.169

The main textural results of the zeolitic waste show that there are similar values in surface area. The S_BET_-specific surface area of Zeo waste 3 is slightly higher (138.89 m^2^/g) than the other two surface areas (137.69 m^2^/g and 137.39 m^2^/g) of Zeo waste 1 and Zeo waste 2, respectively.

After modifying Zeo waste 1 with a hydrogen peroxide solution, S_BET_ remained almost unchanged, but S_Pore_ slightly increased from 138.43 m^2^/g to 143.70 m^2^/g for Zeo waste 1 and Zeo waste 2, respectively (Table 3). This could be related to the H_2_O_2_ solution reacting with the zeolite, which resulted in the removal of carbonaceous impurities and sulfur.

**Figure 3:**
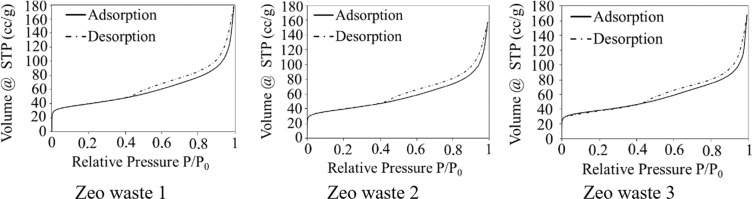
Adsorbption/desorbtion isotherms of N_2_.

According to the International Union of Pure and Applied Chemistry (IUPAC) classification^34^, the obtained isotherms of Zeo waste 1, Zeo waste 2 and Zeo waste 3 (Fig. 3) could be attributed to type I and IV isotherms. A type I isotherm is chosen because of an increasing ratio of *P*/*P*_0_ from 0 to 0.4; thus, the process proceeds according to the type I isotherm, where micropores are predominant. A type IV isotherm occurs when the ratio of *P*/*P*_0_ varies from 0.4 to 1, and according to the type IV isotherm, a mesoporous structure predominates and forms a hysteresis loop. Therefore, the nitrogen isotherms of the samples were a combination of type I at lower relative pressures (*P*/*P*_0_) and type IV at high values of *P*/*P*_0_, suggesting the existence of both micropores and mesopores in the sample.

S. Brunauer, P.H. Emmett and E. Teller in^35^ derived an isotherm labeled as shown in Eq. (3):

3 $$v=\frac{{v}_{m}cP}{({P}_{0}-P)[1+(c-1)(P/{P}_{0})]};$$

where *v* is the volume of adsorbate at some *P*⁄*P*_0_, *v*_*m*_ is the volume of gas adsorbed when the entire adsorbent surface is covered with a complete unimolecular layer, *P* is the gas pressure, *P*_0_ is the saturation pressure of the gas, and *c* is a constant associated with the energy of adsorption.

**Figure 4:**
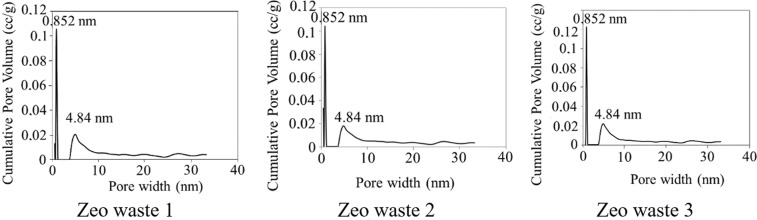
The distribution of the pore structure (PSD DFT slit-cylindric pores model).

The above results are confirmed by calculations using the DFT method. In these materials, micropores with a size of 0.852 nm (diameter) and mesopores with a size of 4.84 nm dominate by using a “slit/cylindric pore, QSDFT adsorption branch” model (Fig. 4). If the “slit pore, QSDFT equilibrium model” is used, mesopores with a size of 3.39 nm dominate. By using a Type I model, the mismatches between the experimental and model curves are 0.537%, 0.641% and 0.652%, and for the second model, they are 0.982, 1.133 and 1.097%, for the Zeo waste 2, Zeo waste 3 and Zeo waste 1 samples, respectively. Therefore, it was interpreted that a “slit/cylinder” model was more fitting to describe the measurement curve. However, if it were based on classical BJH methodology, then according to BJH, the analyzed waste would contain 3.707 nm mesopores, which would then be in closer relation to the “slit pore, QSDFT equilibrium model” calculations of mesopores. Unfortunately, the size of micropores cannot be obtained according to BJH methodology. Nevertheless, with the knowledge that BJH methodology always calculates smaller pore sizes than QSDFT, we state that the “slit/cylinder pores, QSDFT adsorption branch model” best describes the analyzed zeolitic waste.

### Ammonium sorption capacity of zeolitic waste

The ammonium removal capacities of zeolitic waste with different particle sizes were investigated. Adsorption processes in zeolitic materials were performed under two different ammonium concentrations: 1 mg/L and 10 mg/L initial solutions. For the removal of ammonium ions from water solutions, three types of zeolitic waste were chosen.

According to the fundamentals of an ion-exchange reaction^36^ with zeolitic waste, a chemical process involving valence forces is described through the sharing or exchange of electrons between negatively charged zeolite sites and ammonium cations as expressed by using the following equation:

4 $${\rm{Zeolite}}-{{\rm{H}}}_{3}{{\rm{O}}}^{+}+{n}{{\rm{NH}}}_{4}^{+}\leftrightarrow {\rm{Zeolite}}-{n}{{\rm{NH}}}_{4}^{+}+{{\rm{H}}}_{3}{{\rm{O}}}^{+}$$

where H represents exchangeable ions in zeolite and *n* is the number of electric charges.

**Figure 5:**
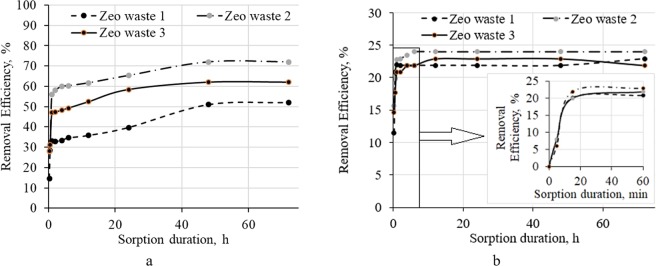
Removal efficiency of NH_4_^+^ ion from aqueous solutions by batch experiments. Initial concentrations: *a* – *C*_0_ = 1 mg/L and *b* – *C*_0_ = 10 mg/L.

**Figure 6:**
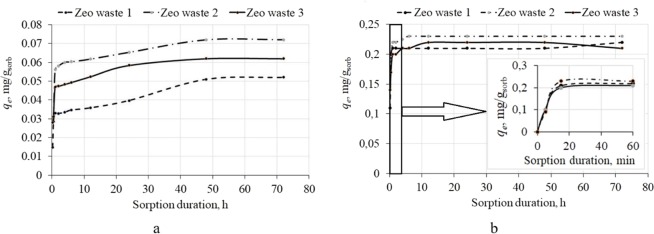
Amounts of exchanged NH_4_^+^ ion *q*_*e*_, mg/g from aqueous solutions by batch experiments. Initial concentrations: *a* – *C*_0_ = 1 mg/L and *b* – *C*_0_ = 10 mg/L.

First, the required time for the sorption equilibrium time was determined. After 1 h of sorption duration, significant ammonium ion sorption was performed (Figs. 5 and 6). During this time, the removal efficiency of NH_4_^+^ ions for Zeo waste 1 was 33%, Zeo waste 3 was 47% and the highest removal efficiency of 56% was reached for Zeo waste 2 when the initial NH_4_^+^ ion concentration was 1 mg/L (Fig. 5a). Equilibrium times were reached in 48 h for all three types of zeolitic waste. After a longer contact time, the removal efficiency gradually increased, and a similar range of zeolitic waste removal efficiency was determined: Zeo waste 2 (72%) > Zeo waste 3 (62%) > Zeo waste 1 (52%). It can be stated that equilibrium times became shorter with an increased initial concentration of NH_4_^+^ (*C*_0_ = 10 mg/L) in the purified solutions (Fig. 5b). Equilibrium times were reached after 15 min in these cases. Removal efficiency reached approximately 23% for all three types of zeolitic waste, and significant differences between the used types of zeolitic waste were not detected. Franus *et al*.^37^ published similar results related to experiments in ammonium ion removal. They stated that the amount of NH_4_^+^ ions removed from aqueous solutions increased with increasing concentrations of NH_4_^+^ ions in the purified solution.

The ion exchange capacity of Zeo waste 2 was 0.072 mg/g; for Zeo waste 3, it was 0.062 mg/g and 0.052 mg/g for Zeo waste 1 when the initial NH_4_^+^ ion concentration was 1 mg/L. When the initial concentration increased to 10 mg/L, higher values (approximately 0.23 mg/g) of exchanged NH_4_^+^ ions were reached, and the amount of exchanged NH_4_^+^ ions was similar for all zeolitic waste.

Based on the experimental results, it can be stated that zeolitic waste with finer particles (Zeo waste 3) has better removal efficiency compared with that of coarser zeolitic waste particles (Zeo waste 1), especially when the initial NH_4_^+^ ion concentration was lower (1 mg/L). Therefore, the ammonium sorption increased with decreasing particle size of the zeolites^32^. This could be related to the ammonium cations having a much easier exchange with smaller zeolite particles that are in the aqueous solution. The BET surface area is calculated with the entire surface area, such as the surface of the pores and the external surface of the material, and is calculated by the adsorption of nitrogen gas on the surface of the materials. The BET surface area is generally higher than the total surface area, which depends on the particle size. Therefore, the BET surface area is not directly dependent on the particle size of zeolites, which is almost the same (Table 3). The best results of ammonium sorption were determined for Zeo waste 2, which was modified with H_2_O_2_. The removal efficiency was 72% after equilibrium was reached.

The initial ammonium concentration is an important aspect in the sorption process of ammonium on zeolite. The adsorption efficiencies are varied at a lower initial concentration (*C*_0_ = 1 mg/L) of ammonia but are almost the same at higher concentrations (*C*_0_ = 10 mg/L) of ammonia (Figs. 5 and 6). This could be explained by the porous microstructure of zeolites. By increasing the initial ammonium concentration, the mass transfer driving force and the rate at which ammonium ions pass to the surface of the zeolite also increased. Therefore, the ammonium ions could transfer from the external surface to the internal micropores of the zeolite^38^. At a lower initial concentration (*C*_0_ = 1 mg/L) of ammonia, ammonium ions could first exchange with cations (H_3_O^+^) on the external surface of the zeolite and only after that it would exchange in the internal surface of the zeolite. For that reason, it is important to determine the particle size and specific surface area of the zeolites, which are related to the sorption process of ammonium (Zeo waste 3 (62%) >Zeo waste 1 (52%)). The best removal efficiency was determined for Zeo waste 2 (72%) because this zeolitic waste was activated with H_2_O_2_ and improved the zeolite sorption capacity.

The pH of an aqueous solution is an important controlling parameter in sorption processes and *water quality* parameters for a fish recirculation system. The removal of NH_4_^+^ ions from an aqueous solution using zeolitic waste was studied at pH 5.5–7.0. The pH decreased in all solutions during all sorption durations because zeolitic waste is highly selective for H_3_O^+^ ions when the H_3_O^+^ ion concentration is higher. Thus, at lower pH values, H_3_O^+^ ions compete with NH_4_^+^ ions for the exchange sites in zeolitic waste samples^39^.

**Figure 7:**
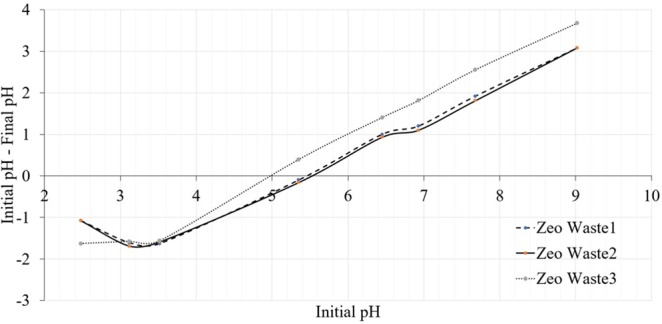
Determination of zero point charge of zeolitic materials according pH drift method.

According to the pH_pzc_ values, it is possible to evaluate the possible attraction and repulsion between zeolitic materials and ammonium in solution. Three types of zeolitic materials were analyzed by evaluating the point of zero charge (pH_pzc_), which was closely related to the sorption properties of the materials. Figure 7 shows the pH drift tests, and all curves have similar shapes. The determined pH_pzc_ values are 5.4 ± 0.1, 5.5 ± 0.1 and 5.0 ± 0.1 for Zeo waste 1, Zeo waste 2 and Zeo waste 3, respectively (Fig. 7). The surfaces of all three samples were positive at a pH lower than the pH_PZC_ and negative at a pH higher than the pH_pzc_.

When the water solution has a high pH, the amount of NH_4_^+^ in solution is very small, and it changes to NH_3_ because the dissociation constant (pKa) of NH_4_^+^ is 9.24^40^. Murayama *et al*.^40^ stated that the recommended pH is from 4 to 10 for removal of NH_4_^+^ in water solution by using zeolites. According to these experimental results, the pH was in the range of 5.5–7.0. The influence of pH values on removal efficiency was not observed to be significant^41^. Therefore, all three types of zeolitic materials have good possibilities for ammonium ion immobilization.

Based on the above results, it can be concluded that synthetic zeolitic waste with two different granulometric compositions and one by using chemical modification with H_2_O_2_ are suitable adsorbents for NH_4_^+^ ion removal and have great potential for removing ammonium ions from water, which can be used in closed fish-farming systems.

### FT-IR analysis of zeolitic waste before and after ammonium ion sorption

**Figure 8:**
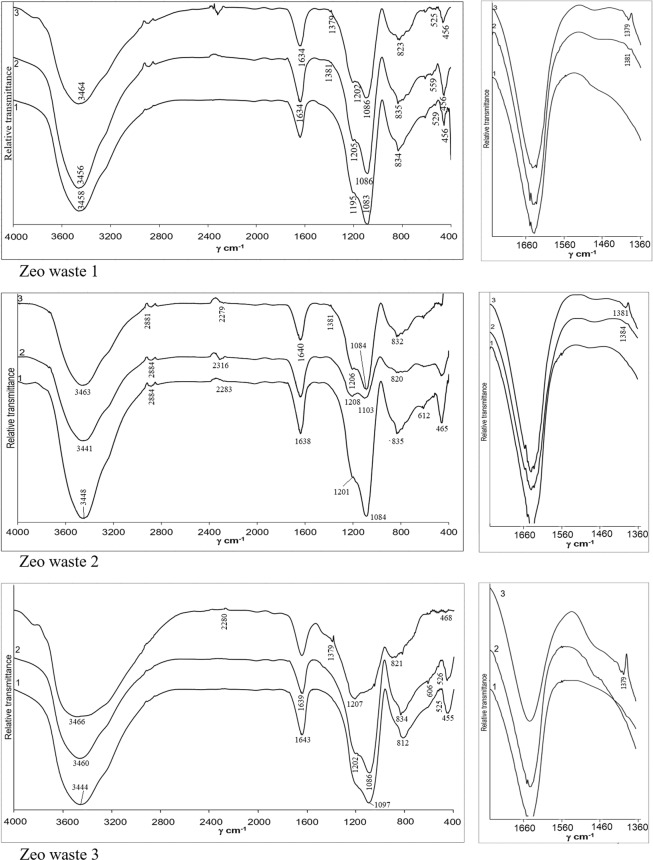
IR spectra of zeolitic waste before ammonium adsorption (1) and after ammonium adsorption (2 and 3). The initial concentration of solutions was 1 mg NH_4_^+^/L and 10 mg NH_4_^+^/L respectively.

After ammonium sorption by zeolitic waste, changes occur in the mineral composition of the zeolitic waste, which was evaluated by using FT-IR analysis. Figure 8 presents the IR spectra of the three types of zeolitic waste before and after ammonium adsorption experiments. Two initial solution concentrations of 1 mg/L NH_4_^+^ and 10 mg/L NH_4_^+^ were used. All three types of zeolitic waste before sorption have similar characteristics. The IR spectrum of zeolitic waste samples showed broad absorption bands at 3441–3466 cm^−1^ and 1634–1643 cm^−1^ attributed to the OH stretching vibration and usual H-O-H bending vibration, respectively^42^. The broad absorption bands at approximately 1083–1103 cm^−1^ were attributed to the asymmetrical stretching vibration of Si-O-Si and Si-O-Al in a tetrahedral. Absorption bands at 820 cm^−1^–835 cm^−1^ were assigned to Si–O–Si symmetrical stretching. The peaks at 455 cm^−1^–465 cm^−1^ were assigned to tetrahedral vibration. The weak peaks at 525 cm^−1^–559 cm^−1^ were related to a double ring external linkage related to an FAU structure. Sang *et al*.^43^ also reported that all of the abovementioned absorption bands were typical for zeolite Y. These FT-IR peaks agreed well with the XRD patterns (Fig. 1).

It was obvious that the sorption process does not change the main mineral composition of zeolitic waste, but one new band is observed after NH_4_^+^ sorption. In these cases, the initial solution concentration was 10 mg/L NH_4_^+^. Changes occurred during the sorption process and were related to the additional absorption frequencies of ammonium. The results confirmed that the main changes were observed at approximately 1389–1384 cm^−1^, attributing to the absorption band of NH_4_^+^ present in the zeolite structure^44^. The normal modes of vibration of a free NH_4_^+^ ion have frequencies of 1389–1384 cm^−1^. When using a lower initial solution concentration of 1 mg/L NH_4_^+^, the bands related to NH^4+^ ion frequencies were weaker compared with that of zeolitic waste materials with a higher initial solution concentration (10 mg/L NH_4_^+^) (Fig. 7).

In conclusion, it is evident that the most efficient zeolitic waste for ammonium ion sorption is Zeo waste 2. The investigated zeolitic waste materials could be an excellent material to remove ammonium impurities from water and wastewater.

## Conclusion

- After H_2_O_2_ modification of zeolitic waste (Zeo waste 2), the amount of carbon (technically, coke) significantly decreased from 3.31% in unmodified zeolitic waste to 1.25% in H_2_O_2_-treated zeolitic waste. Sulfur was not detected at all for the oxidation reaction. This modification did not change the mineral composition of zeolitic waste. According to XRD analysis, the zeolitic waste is composed of zeolite Y.
- All three types of zeolitic waste powders were spherical particles. According to an analysis of nitrogen adsorption-desorption isotherms, zeolitic waste demonstrated a low specific surface area SBET (approximately 138 m^2^/g) and combined micropores (0.852 nm) and mesopores (4.84 nm). All three types of zeolitic waste can be classified as microporous materials with mesopores present in them. Based on the results of the study, the pHpzc values of Zeo waste 1, Zeo waste 2 and Zeo waste 3 were 5.4 ± 0.1, 5.5 ± 0.1 and 5.0 ± 0.1, respectively.
- The maximal ammonium ion removal efficiency of 72% was for Zeo waste 2 (zeolitic waste modified with H_2_O_2_) when the initial NH_4_^+^ ion concentration was 1 mg/L. This could be related to the highest pore surface area of 143.70 m^2^/g compared with those of the other two types of investigated zeolitic waste. When the initial NH_4_^+^ ion concentration was higher (10 mg/L), equilibrium times were reached after 15 min, and the removal efficiency was approximately 23% for all three types of zeolitic waste.
- In this study, experimental results showed that zeolitic waste materials are suitable adsorbents for the removal of NH_4_^+^ ion impurities from water and wastewater. In our experimental conditions, the maximum removal efficiency was 72% for NH_4_^+^ ions.
